# Circadian‐regulating nuclei in the human anterior hypothalamus demonstrate selective vulnerability to pathological effects of Alzheimer's disease

**DOI:** 10.1002/alz70855_107235

**Published:** 2025-12-25

**Authors:** Grace Judge, Gowoon Son, Mihovil Mladinov, Felipe Luiz Pereira, Tia LaMore, Yuheng Chen, Lucile Zhu, Yishu Chao, Song Hua Li, Claudia Kimie Suemoto, Renata Elaine Paraizo Leite, Vitor Ribeiro Paes, Carlos Augusto Pasqualucci, Wilson Jacob‐Filho, Salvatore Spina, William W. Seeley, Thomas C. Neylan, Lea T. Grinberg

**Affiliations:** ^1^ Memory and Aging Center, UCSF Weill Institute for Neurosciences, University of California, San Francisco, San Francisco, CA, USA; ^2^ University of California, Berkeley, Berkeley, CA, USA; ^3^ Physiopathology in Aging Laboratory (LIM‐22), Department of Internal Medicine, University of São Paulo Medical School, São Paulo, São Paulo, Brazil; ^4^ Division of Geriatrics, Department of Internal Medicine, University of São Paulo Medical School, São Paulo, São Paulo, Brazil; ^5^ Department of Pathology, University of São Paulo Medical School, São Paulo, São Paulo, Brazil; ^6^ Department of Neurology, Memory and Aging Center, University of California San Francisco, San Francisco, CA, USA; ^7^ Department of Psychiatry, University of California, San Francisco, San Francisco, CA, USA

## Abstract

**Background:**

Selective neuronal vulnerability is an underappreciated but critical factor in understanding Alzheimer's disease (AD) pathogenesis, disease progression, and neuroprotective mechanisms. Given the unique characteristics of the human brain, studying selective vulnerability in postmortem human tissue offers invaluable insights. Our group has demonstrated a direct link between subcortical neuronal degeneration in sleep‐wake and circadian‐modulating regions and clinical function, highlighting these structures as key to investigating selective vulnerability. Circadian control is primarily governed by the anterior hypothalamus, where the suprachiasmatic nucleus (SCN) acts as the brain's master clock. The SCN and neighboring structures, including the paraventricular nucleus (PVN) and supraoptic nucleus (SON), share a predominant population of arginine vasopressin‐expressing (AVP+) neurons, which are integral to circadian function. To assess whether these three nuclei provide a platform to study selective vulnerability in AD, we quantified neuronal populations and AD pathology burden in postmortem human brains.

**Method:**

We analyzed postmortem anterior hypothalamic tissue, including the SCN, PVN, and SON, from 12 controls (Braak stage 0) and 28 AD cases spanning mild to severe pathology (Braak stages I, II, and VI). Using fluorescence in situ hybridization, we probed AVP+ neurons in these nuclei and quantified neuronal estimates and AVP+ neuronal area. A custom 2D image registration method was used to quantify amyloid plaques and neurofibrillary tangles in adjacent sections.

**Result:**

AVP+ neuronal loss was evident in the SCN as early as Braak stage II, whereas AVP+ neurons remained stable in the PVN and SON. Both the SCN and PVN exhibited phosphorylated tau inclusions, while amyloid plaques were detected in the PVN but absent in the SCN and SON. The SON displayed no evidence of amyloid plaques or tau accumulation, suggesting resistance to AD pathology.

**Conclusion:**

Our findings reveal that among these three AVP+ nuclei, the SCN is particularly vulnerable to early AD‐related neurodegeneration, whereas the PVN accumulates tau and amyloid pathology without significant neuronal loss. The SON appears relatively resistant to AD pathology. These differential patterns suggest that intrinsic properties of neuronal populations and local microenvironments may contribute to selective vulnerability, providing a unique model to explore resilience mechanisms in AD.

